# 2-[*N*-(3-Amino-4-nitro­phen­yl)carboximido­yl]phenol

**DOI:** 10.1107/S160053681104181X

**Published:** 2011-10-12

**Authors:** Gholam Hossein Shahverdizadeh, Seik Weng Ng, Edward R. T. Tiekink, Babak Mirtamizdoust

**Affiliations:** a Department of Chemistry, Faculty of Science, Tabriz Branch, Islamic Azad University, Tabriz, PO Box 1655, Iran; b Department of Chemistry, University of Malaya, 50603 Kuala Lumpur, Malaysia; c Chemistry Department, Faculty of, Science, King Abdulaziz University, PO Box 80203 Jeddah, Saudi Arabia; d Department of Inorganic Chemistry, Faculty of Chemistry, University of Tabriz, Tabriz, Iran

## Abstract

The title compound, C_13_H_11_N_3_O_3_, is essentially planar (r.m.s. for the 19 non-H atoms = 0.031 Å), a conformation stabilized in part by intra­molecular O—H⋯N and N—H⋯O hydrogen bonds. The configuration about the imine bond [1.2919 (12) Å] is *E*. The presence of N—H⋯O(nitro) hydrogen bonds leads to the formation of supra­molecular tapes in the crystal structure. These are connected into layers by π–π inter­actions [centroid–centroid distance = 3.6046 (6) Å] occurring between the hy­droxy- and amino-substituted benzene rings.

## Related literature

For related work on Schiff bases, see: Prasath *et al.* (2010 ▶); Shahverdizadeh & Tiekink (2011 ▶). For specialized crystallization techniques, see: Harrowfield *et al.* (1996 ▶).
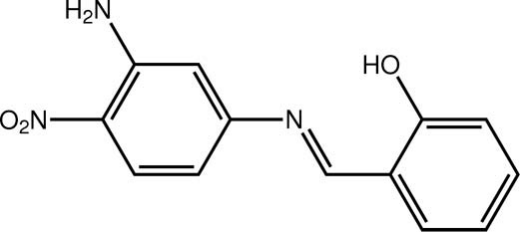


## Experimental

### 

#### Crystal data

C_13_H_11_N_3_O_3_
*M* *_r_* = 257.25
Triclinic, 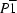

*a* = 7.0961 (3) Å
*b* = 7.5168 (4) Å
*c* = 12.1627 (6) Å
α = 100.067 (4)°
β = 94.751 (4)°
γ = 115.011 (5)°
*V* = 569.87 (5) Å^3^
*Z* = 2
Cu *K*α radiation
μ = 0.92 mm^−1^
*T* = 100 K
0.25 × 0.20 × 0.15 mm

#### Data collection

Agilent SuperNova Dual diffractometer with an Atlas detector
Absorption correction: multi-scan (*CrysAlis PRO*; Agilent, 2010 ▶) *T*_min_ = 0.748, *T*_max_ = 1.000
3654 measured reflections
2231 independent reflections
2105 reflections with *I* > 2σ(*I*)
*R*_int_ = 0.008

#### Refinement

*R*[*F* ^2^ > 2σ(*F* ^2^)] = 0.035
*wR*(*F* ^2^) = 0.113
*S* = 1.07
2231 reflections
184 parameters
3 restraints
H atoms treated by a mixture of independent and constrained refinement
Δρ_max_ = 0.28 e Å^−3^
Δρ_min_ = −0.28 e Å^−3^

### 

Data collection: *CrysAlis PRO* (Agilent, 2010 ▶); cell refinement: *CrysAlis PRO*; data reduction: *CrysAlis PRO*; program(s) used to solve structure: *SHELXS97* (Sheldrick, 2008 ▶); program(s) used to refine structure: *SHELXL97* (Sheldrick, 2008 ▶); molecular graphics: *ORTEP-3* (Farrugia, 1997 ▶) and *DIAMOND* (Brandenburg, 2006 ▶); software used to prepare material for publication: *publCIF* (Westrip, 2010 ▶).

## Supplementary Material

Crystal structure: contains datablock(s) global, I. DOI: 10.1107/S160053681104181X/hg5110sup1.cif

Structure factors: contains datablock(s) I. DOI: 10.1107/S160053681104181X/hg5110Isup2.hkl

Additional supplementary materials:  crystallographic information; 3D view; checkCIF report

## Figures and Tables

**Table 1:** Hydrogen-bond geometry (Å, °)

*D*—H⋯*A*	*D*—H	H⋯*A*	*D*⋯*A*	*D*—H⋯*A*
O1—H1*o*⋯N1	0.86 (1)	1.79 (1)	2.5933 (10)	154 (2)
N2—H1*n*⋯O2	0.89 (1)	2.06 (1)	2.6542 (11)	123 (1)
N2—H1*n*⋯O2^i^	0.89 (1)	2.42 (1)	3.1479 (11)	140 (1)
N2—H2*n*⋯O3^ii^	0.86 (1)	2.25 (1)	3.0746 (10)	161 (1)

Symmetry codes: (i) 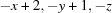; (ii) 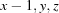.

**Fig. 1.**
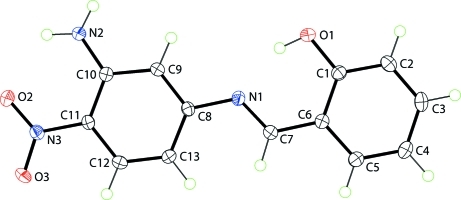
The molecular structure of (I) showing the atom-labelling scheme and displacement ellipsoids at the 50% probability level.

**Fig. 2.**
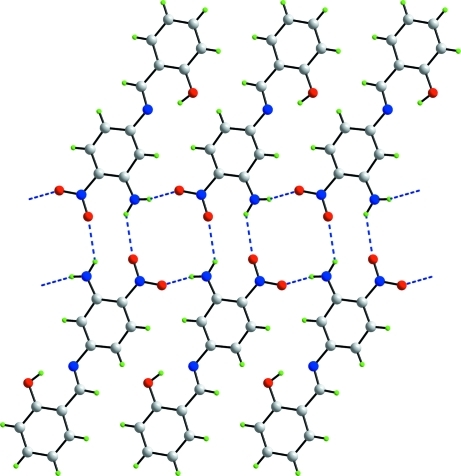
A view of the supramolecular tape in the (1 1 1) plane and sustained by N—H···O hydrogen bonds (blue dashed lines) in the crystal structure of (I).

**Fig. 3.**
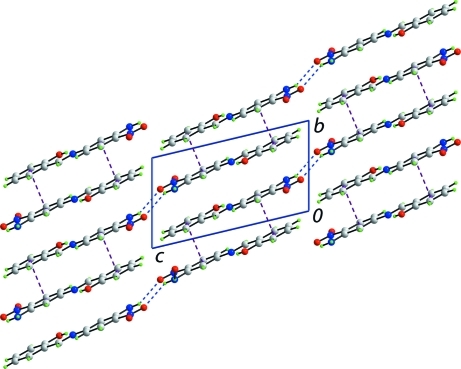
A view in projection down the a axis of the crystal packing in (I) highlighting the stacking of layers. The N—H···O and π–π interactions are shown as blue and purple dashed lines, respectively.

## supplementary crystallographic information

### Comment

In continuation of structural studies of Schiff bases (Prasath *et al.*, 2010; Shahverdizadeh & Tiekink, 2011), the title compound was synthesized and characterized crystallographically.

The molecule of (I), Fig. 1, is planar with the r.m.s. deviation of the 19 non-hydrogen atoms being 0.031 Å; the maximum and minimum deviations are 0.066 (1) Å for atom C12 and -0.062 (1) Å for atom O2. The observed planar conformation is stabilized in part by intramolecular O—H···N and N—H···O hydrogen bonds, Table 1. The amino group is planar with the sum of the angles about the N2 atom being approximately 359°. The configuration about the N1═ C7 bond [1.2919 (12) Å] is *E*, and the hydroxy and amino groups are *syn*.

In the crystal structure, supramolecular tapes in the (1 1 1) plane are formed *via N*—*H*···*O*(nitro) hydrogen bonds, Fig. 2 and Table 1. The spine of the tape comprises alternating 12-membered rectangular {···HNC_2_NO}_2_ and square {···HNH···ONO}_2_ synthons. The tapes are connected into layers *via*π–π interactions occurring between the hydroxy- and amino-benzene rings [centroid(C1–C6)···centroid(C8–C13)^i^ distance = 3.6046 (6) Å for *i*: 1 - *x*, -*y*, 1 - *z*].

### Experimental

A solution of 4-nitrobenzene-1,3-diamine (10 mmol) in methanol (50 ml) was added drop wise to a solution of salicylaldehyde (10 mmol) in methanol (50 ml). The mixture was stirred for 5 h. The resulting solution was filtered to obtain a Schiff base, and dried. Single crystals of the title compound were obtained by using the branched tube method (Harrowfield *et al.*, 1996). Thus, the Schiff base (5 mmol) was placed in the arm to be heated. Methanol was added to fill both arms, and then the arm to be heated was placed in a bath at 333 K. After 2 days, orange crystals were deposited in the cooler arm, which were filtered, washed with water and air dried. Yield: 88%. *M*.pt.: 432 K.

### Refinement

Carbon-bound H-atoms were placed in calculated positions [C—H 0.95 Å, *U*_iso_(H) = 1.2*U*_eq_(C)] and were included in the refinement in the riding model approximation. The O—H and N—H H atoms were located from a difference map and refined with O—H = 0.84±0.01 Å and N—H = 0.88±0.01 Å, respectively, and with unconstrained *U_iso_*(H) values.

### Crystal data

**Table:**

C_13_H_11_N_3_O_3_	*Z* = 2
*M**_r_* = 257.25	*F*(000) = 268
Triclinic, *P*1	*D*_x_ = 1.499 Mg m^−^^3^
Hall symbol: -P 1	Cu *K*α radiation, λ = 1.54184 Å
*a* = 7.0961 (3) Å	Cell parameters from 2525 reflections
*b* = 7.5168 (4) Å	θ = 3.8–74.1°
*c* = 12.1627 (6) Å	µ = 0.92 mm^−^^1^
α = 100.067 (4)°	*T* = 100 K
β = 94.751 (4)°	Prism, orange
γ = 115.011 (5)°	0.25 × 0.20 × 0.15 mm
*V* = 569.87 (5) Å^3^	

### Data collection

**Table:**

Agilent SuperNova Dual diffractometer with an Atlas detector	2231 independent reflections
Radiation source: SuperNova (Cu) X-ray Source	2105 reflections with *I* > 2σ(*I*)
Mirror	*R*_int_ = 0.008
Detector resolution: 10.4041 pixels mm^-1^	θ_max_ = 74.3°, θ_min_ = 3.8°
ω scans	*h* = −8→8
Absorption correction: multi-scan (*CrysAlis PRO*; Agilent, 2010)	*k* = −9→9
*T*_min_ = 0.748, *T*_max_ = 1.000	*l* = −13→15
3654 measured reflections	

### Refinement

**Table:**

Refinement on *F*^2^	Primary atom site location: structure-invariant direct methods
Least-squares matrix: full	Secondary atom site location: difference Fourier map
*R*[*F*^2^ > 2σ(*F*^2^)] = 0.035	Hydrogen site location: inferred from neighbouring sites
*wR*(*F*^2^) = 0.113	H atoms treated by a mixture of independent and constrained refinement
*S* = 1.07	*w* = 1/[σ^2^(*F*_o_^2^) + (0.0753*P*)^2^ + 0.0839*P*] where *P* = (*F*_o_^2^ + 2*F*_c_^2^)/3
2231 reflections	(Δ/σ)_max_ < 0.001
184 parameters	Δρ_max_ = 0.28 e Å^−^^3^
3 restraints	Δρ_min_ = −0.28 e Å^−^^3^

### Special details

**Table:**

Geometry. All e.s.d.'s (except the e.s.d. in the dihedral angle between two l.s. planes) are estimated using the full covariance matrix. The cell e.s.d.'s are taken into account individually in the estimation of e.s.d.'s in distances, angles and torsion angles; correlations between e.s.d.'s in cell parameters are only used when they are defined by crystal symmetry. An approximate (isotropic) treatment of cell e.s.d.'s is used for estimating e.s.d.'s involving l.s. planes.
Refinement. Refinement of *F*^2^ against ALL reflections. The weighted *R*-factor *wR* and goodness of fit *S* are based on *F*^2^, conventional *R*-factors *R* are based on *F*, with *F* set to zero for negative *F*^2^. The threshold expression of *F*^2^ > σ(*F*^2^) is used only for calculating *R*-factors(gt) *etc*. and is not relevant to the choice of reflections for refinement. *R*-factors based on *F*^2^ are statistically about twice as large as those based on *F*, and *R*- factors based on ALL data will be even larger.

### Fractional atomic coordinates and isotropic or equivalent isotropic displacement parameters (Å^2^)

**Table:**

	*x*	*y*	*z*	*U*_iso_*/*U*_eq_	
O1	0.40452 (11)	0.27962 (11)	0.59127 (6)	0.0227 (2)	
H1o	0.495 (2)	0.285 (3)	0.5475 (13)	0.062 (5)*	
O2	1.09653 (11)	0.36753 (12)	0.05261 (6)	0.0246 (2)	
O3	1.31365 (10)	0.29051 (11)	0.14605 (6)	0.0233 (2)	
N1	0.71614 (12)	0.25239 (11)	0.50498 (7)	0.0158 (2)	
N2	0.78293 (13)	0.40302 (13)	0.14327 (7)	0.0191 (2)	
H1n	0.840 (2)	0.420 (2)	0.0816 (9)	0.039 (4)*	
H2n	0.6610 (15)	0.4021 (18)	0.1447 (10)	0.026 (3)*	
N3	1.15808 (12)	0.32599 (12)	0.13890 (7)	0.0172 (2)	
C1	0.47038 (15)	0.22517 (14)	0.68163 (8)	0.0168 (2)	
C2	0.36032 (15)	0.21048 (14)	0.77211 (8)	0.0197 (2)	
H2	0.2408	0.2372	0.7690	0.024*	
C3	0.42483 (16)	0.15713 (14)	0.86643 (8)	0.0209 (2)	
H3	0.3500	0.1491	0.9281	0.025*	
C4	0.59855 (15)	0.11503 (14)	0.87207 (8)	0.0202 (2)	
H4	0.6405	0.0763	0.9366	0.024*	
C5	0.70904 (15)	0.13020 (14)	0.78289 (8)	0.0182 (2)	
H5	0.8283	0.1031	0.7870	0.022*	
C6	0.64775 (14)	0.18514 (13)	0.68626 (8)	0.0155 (2)	
C7	0.76768 (14)	0.20111 (13)	0.59454 (8)	0.0161 (2)	
H7	0.8864	0.1733	0.6004	0.019*	
C8	0.83502 (14)	0.27202 (13)	0.41602 (8)	0.0146 (2)	
C9	0.76291 (14)	0.32620 (13)	0.32488 (8)	0.0151 (2)	
H9	0.6412	0.3491	0.3265	0.018*	
C10	0.86365 (14)	0.34913 (13)	0.22862 (8)	0.0150 (2)	
C11	1.04545 (14)	0.31485 (13)	0.23212 (8)	0.0150 (2)	
C12	1.12150 (14)	0.26527 (14)	0.32711 (8)	0.0168 (2)	
H12	1.2463	0.2472	0.3281	0.020*	
C13	1.01929 (15)	0.24257 (14)	0.41788 (8)	0.0172 (2)	
H13	1.0711	0.2076	0.4812	0.021*	

### Atomic displacement parameters (Å^2^)

**Table:**

	*U*^11^	*U*^22^	*U*^33^	*U*^12^	*U*^13^	*U*^23^
O1	0.0209 (4)	0.0344 (4)	0.0206 (4)	0.0173 (3)	0.0055 (3)	0.0120 (3)
O2	0.0274 (4)	0.0366 (4)	0.0191 (4)	0.0190 (3)	0.0095 (3)	0.0143 (3)
O3	0.0206 (4)	0.0328 (4)	0.0259 (4)	0.0180 (3)	0.0105 (3)	0.0104 (3)
N1	0.0162 (4)	0.0170 (4)	0.0136 (4)	0.0067 (3)	0.0031 (3)	0.0038 (3)
N2	0.0185 (4)	0.0274 (4)	0.0183 (4)	0.0143 (3)	0.0067 (3)	0.0102 (3)
N3	0.0166 (4)	0.0173 (4)	0.0184 (4)	0.0077 (3)	0.0051 (3)	0.0047 (3)
C1	0.0167 (5)	0.0163 (4)	0.0164 (5)	0.0068 (4)	0.0018 (4)	0.0036 (3)
C2	0.0183 (5)	0.0190 (4)	0.0219 (5)	0.0088 (4)	0.0054 (4)	0.0028 (4)
C3	0.0241 (5)	0.0193 (5)	0.0166 (5)	0.0066 (4)	0.0082 (4)	0.0032 (4)
C4	0.0240 (5)	0.0201 (4)	0.0146 (5)	0.0079 (4)	0.0023 (4)	0.0052 (3)
C5	0.0182 (5)	0.0179 (5)	0.0181 (5)	0.0076 (4)	0.0023 (4)	0.0048 (3)
C6	0.0157 (4)	0.0141 (4)	0.0149 (5)	0.0054 (3)	0.0019 (3)	0.0027 (3)
C7	0.0153 (5)	0.0160 (4)	0.0170 (5)	0.0071 (4)	0.0031 (4)	0.0036 (4)
C8	0.0135 (4)	0.0140 (4)	0.0146 (5)	0.0049 (3)	0.0030 (3)	0.0021 (3)
C9	0.0140 (4)	0.0157 (4)	0.0163 (5)	0.0073 (3)	0.0030 (4)	0.0033 (4)
C10	0.0153 (4)	0.0132 (4)	0.0153 (5)	0.0055 (3)	0.0025 (3)	0.0031 (3)
C11	0.0148 (4)	0.0153 (4)	0.0151 (5)	0.0063 (4)	0.0046 (4)	0.0039 (3)
C12	0.0138 (4)	0.0179 (4)	0.0195 (5)	0.0079 (4)	0.0025 (3)	0.0038 (3)
C13	0.0171 (4)	0.0207 (5)	0.0152 (5)	0.0094 (4)	0.0019 (3)	0.0056 (3)

### Geometric parameters (Å, °)

**Table:**

O1—C1	1.3519 (11)	C4—C5	1.3830 (13)
O1—H1o	0.858 (9)	C4—H4	0.9500
O2—N3	1.2424 (10)	C5—C6	1.4064 (13)
O3—N3	1.2403 (10)	C5—H5	0.9500
N1—C7	1.2919 (12)	C6—C7	1.4494 (13)
N1—C8	1.4163 (12)	C7—H7	0.9500
N2—C10	1.3471 (12)	C8—C9	1.3772 (13)
N2—H1n	0.885 (9)	C8—C13	1.4145 (13)
N2—H2n	0.864 (8)	C9—C10	1.4200 (12)
N3—C11	1.4346 (12)	C9—H9	0.9500
C1—C2	1.3947 (14)	C10—C11	1.4172 (13)
C1—C6	1.4115 (14)	C11—C12	1.4079 (13)
C2—C3	1.3840 (14)	C12—C13	1.3668 (13)
C2—H2	0.9500	C12—H12	0.9500
C3—C4	1.3957 (15)	C13—H13	0.9500
C3—H3	0.9500		
			
C1—O1—H1o	104.2 (12)	C5—C6—C7	119.76 (9)
C7—N1—C8	121.52 (8)	C1—C6—C7	121.50 (9)
C10—N2—H1n	122.2 (10)	N1—C7—C6	121.24 (9)
C10—N2—H2n	117.9 (8)	N1—C7—H7	119.4
H1n—N2—H2n	119.2 (12)	C6—C7—H7	119.4
O3—N3—O2	121.55 (8)	C9—C8—C13	120.23 (9)
O3—N3—C11	118.76 (8)	C9—C8—N1	115.89 (8)
O2—N3—C11	119.68 (8)	C13—C8—N1	123.87 (8)
O1—C1—C2	118.68 (9)	C8—C9—C10	122.45 (8)
O1—C1—C6	121.45 (9)	C8—C9—H9	118.8
C2—C1—C6	119.87 (9)	C10—C9—H9	118.8
C3—C2—C1	120.17 (9)	N2—C10—C11	125.47 (8)
C3—C2—H2	119.9	N2—C10—C9	118.58 (8)
C1—C2—H2	119.9	C11—C10—C9	115.95 (8)
C2—C3—C4	120.79 (9)	C12—C11—C10	121.18 (9)
C2—C3—H3	119.6	C12—C11—N3	117.08 (8)
C4—C3—H3	119.6	C10—C11—N3	121.73 (8)
C5—C4—C3	119.36 (9)	C13—C12—C11	121.27 (9)
C5—C4—H4	120.3	C13—C12—H12	119.4
C3—C4—H4	120.3	C11—C12—H12	119.4
C4—C5—C6	121.07 (9)	C12—C13—C8	118.89 (9)
C4—C5—H5	119.5	C12—C13—H13	120.6
C6—C5—H5	119.5	C8—C13—H13	120.6
C5—C6—C1	118.73 (9)		
			
O1—C1—C2—C3	−179.34 (8)	N1—C8—C9—C10	−179.14 (7)
C6—C1—C2—C3	0.12 (15)	C8—C9—C10—N2	−179.84 (8)
C1—C2—C3—C4	−0.76 (15)	C8—C9—C10—C11	−0.63 (14)
C2—C3—C4—C5	1.05 (15)	N2—C10—C11—C12	177.90 (8)
C3—C4—C5—C6	−0.70 (15)	C9—C10—C11—C12	−1.25 (13)
C4—C5—C6—C1	0.07 (14)	N2—C10—C11—N3	−3.06 (15)
C4—C5—C6—C7	179.57 (8)	C9—C10—C11—N3	177.79 (7)
O1—C1—C6—C5	179.66 (7)	O3—N3—C11—C12	0.51 (13)
C2—C1—C6—C5	0.22 (14)	O2—N3—C11—C12	179.41 (7)
O1—C1—C6—C7	0.17 (15)	O3—N3—C11—C10	−178.57 (8)
C2—C1—C6—C7	−179.27 (8)	O2—N3—C11—C10	0.32 (13)
C8—N1—C7—C6	178.78 (7)	C10—C11—C12—C13	1.95 (14)
C5—C6—C7—N1	−179.59 (7)	N3—C11—C12—C13	−177.14 (8)
C1—C6—C7—N1	−0.10 (15)	C11—C12—C13—C8	−0.71 (14)
C7—N1—C8—C9	179.58 (7)	C9—C8—C13—C12	−1.16 (14)
C7—N1—C8—C13	−1.46 (15)	N1—C8—C13—C12	179.92 (8)
C13—C8—C9—C10	1.85 (14)		

### Hydrogen-bond geometry (Å, °)

**Table:**

*D*—H···*A*	*D*—H	H···*A*	*D*···*A*	*D*—H···*A*
O1—H1o···N1	0.86 (1)	1.79 (1)	2.5933 (10)	154.(2)
N2—H1n···O2	0.89 (1)	2.06 (1)	2.6542 (11)	123.(1)
N2—H1n···O2^i^	0.89 (1)	2.42 (1)	3.1479 (11)	140.(1)
N2—H2n···O3^ii^	0.86 (1)	2.25 (1)	3.0746 (10)	161.(1)

Symmetry codes: (i) −*x*+2, −*y*+1, −*z*; (ii) *x*−1, *y*, *z*.
